# Platinum catalyzed hydrodeoxygenation of guaiacol in illumination of cresol production: a density functional theory study

**DOI:** 10.1098/rsos.170650

**Published:** 2017-11-08

**Authors:** Anand Mohan Verma, Nanda Kishore

**Affiliations:** Department of Chemical Engineering, Indian Institute of Technology Guwahati, Guwahati, Assam 781039, India

**Keywords:** bio-oil, guaiacol, cresol, hydrodeoxygenation, kinetics, reaction pathway

## Abstract

The unprocessed bio-oil obtained by the pyrolysis of lignocellulosic biomass comprises hundreds of oxy-components which vitiate its quality in terms of low heating value, low stability, low pH, etc. Therefore, it has to be upgraded prior to its use as transportation fuel. In this work, guaiacol, a promising compound of the phenolic fraction of unprocessed bio-oil, is considered as a model component for studying its hydrodeoxygenation over a Pt_3_ catalyst cluster. The production of catechol, 3-methylcatechol, *m*-cresol and *o*-cresol from guaiacol over a Pt_3_ cluster is numerically investigated using density functional theory. Further, the kinetic parameters are obtained over a wide range of temperature, i.e. 473–673 K at an interval of 50 K. Briefly, results indicate that O─H and C─H bond scissions determine the reaction rates of ‘guaiacol to catechol’ and ‘catechol to 3-methylcatechol’ reactions with activation energies of 30.32 and 41.3 kcal mol^−1^, respectively. On the other hand, C─O bond scissions determine the rates of 3-methylcatechol to *m*- and *o*-cresol production reactions, respectively. The kinetics of all reactions indicate that ln *k* versus 1/*T* plots are linear over the entire range of temperature considered herein.

## 1. Introduction

With rapid depletion of conventional energy resources, tremendous increase in the production and use of renewable fuels is foreseeable. Moreover, the pollution created by fossil resources is a big concern across the globe. Nevertheless, various renewable energy resources available, e.g. tidal energy, solar energy, geothermal energy, wind energy, biomass, etc., performing at their peak are balancing the present energy demand to a good extent. Out of all renewable energy resources, only lignocellulosic biomass is the promising renewable energy resource that has the potential of a sustainable carbon element [1]. There are mainly three fractions in lignocellulosic biomass, lignin, cellulose and hemicellulose; however, the lignin fraction of biomass is attracting considerable attention because it can be acquired as a by-product from, for instance, pulp and paper industries, bioethanol processes, etc. [1,2]. In addition, it possesses high energy density compared to the cellulose and hemicellulose fractions [2]. There are many thermochemical methods for the conversion of lignocellulosic biomass, such as pyrolysis, liquefaction and gasification, and pyrolysis is viewed to be the most advantageous compared to the others [3,4]. However, the bio-oil derived from pyrolysis or liquefaction of lignocellulosic biomass consists of a huge number of oxy-functionals which degrade its quality in terms of stability, heating value, low pH, high viscosity, etc.; therefore, it cannot be used directly as transportation fuel [4–6]. Consequently, it has to be upgraded by cleaving the C─O bonds of oxy-components using promising routes. Hydrodeoxygenation (HDO) is viewed to be one of the most promising routes to cleave the C─O bonds with the aid of a hydrogen molecule in the presence of suitable catalysts [2,7]. Nonetheless, the unprocessed bio-oil is a great source for obtaining various platform chemicals, e.g. levulinic acid, hydroxymethyl furfural, cyclohexanol, etc.

The hundreds of oxy-functionals present in bio-oil can be grouped as acids, esters, sugars, phenols, etc. Guaiacol (also known as 2-methoxyphenol) is one of the best representative model compound of phenolic fractions of bio-oil because it comprises two oxy-functionals, namely methoxy and hydroxyl groups [8–12]. Also, guaiacol is one of the most dominant compounds present in many pyrolytic products of lignin [13–16]. Therefore, in this numerical study, guaiacol is considered as a model compound and various possible reactions were carried out to produce catechol, 3-methylcatechol (referred to as methylcatechol henceforth), *m*- and *o*-cresol in the presence of Pt_3_ catalyst within the density functional theory (DFT) framework.

HDO of guaiacol has been considered both experimentally and computationally by several researchers [8–10,17–21]. For instance, Lee *et al*. [10] reported HDO of guaiacol over Pt(111) catalyst and observed that guaiacol undergoes dehydrogenation of the methoxy group instead of direct deoxygenation via demethylation, dehydroxylation and demethoxylation. Lu *et al*. [11,17] studied the HDO of guaiacol over Ru(0001) and Pt(111) catalysts, respectively, and reported the microkinetic modelling over various temperatures. Ru(0001) catalyst produced phenol as the major product, whereas, Pt(111) catalyst produced catechol as the major product. Bykova *et al*. [19] performed experiments on HDO of guaiacol over Ni catalyst and proposed several reaction routes. They reported cyclohexane as the product along with several intermediates such as catechol, phenol, benzene, cyclohexanone and cyclohexanol. Gao *et al*. [20] studied the HDO of guaiacol over Pt/C catalyst experimentally and over Pt(111) catalyst computationally. They reported the formation of catechol, phenol and cyclopentanone as end products. Their analysis showed that conversions of guaiacol to catechol and phenol are first-order reactions. Chiu *et al*. [22] performed computational study of the guaiacol HDO over Ru(0001) catalyst and reported benzene as the final product, with catechol and phenol being intermediate products. Liu *et al*. [23] carried out a non-catalytic guaiacol pyrolysis study using DFT and reported the formation of *o*-quinonemethide, phenol, etc. Leiva *et al*. [24] performed an experimental study on HDO of guaiacol over ReS_2_/SiO_2_ and ReO*_x_*/SiO_2_ catalysts. They reported the formation of methylphenol and methylcyclohexane, and the respective kinetic parameters were discussed. Similarly, Tran *et al*. [25] carried out experiments on HDO of guaiacol over Al-MCM-41 supported Ni and Co catalysts and reported benzene, phenol and cresol as the major products. On the other hand, Yong & Yukihiko [26] carried out an experimental kinetic analysis for guaiacol conversion in sub- and super-critical water. They reported a variety of products including the production of cresol, and reported the overall guaiacol decomposition as a first-order reaction. Saidi *et al*. [2] reviewed characteristics of numerous catalysts for HDO of bio-oil components and concluded that noble metal catalysts are much more suitable for HDO reactions compared to sulfided metal catalysts because HDO of bio-oil components produces water, and sulfided metal catalysts show adverse effect in the aqueous phase milieu whereas the noble metal catalysts perform excellently in both gaseous and aqueous phases.

**Figure 1.**
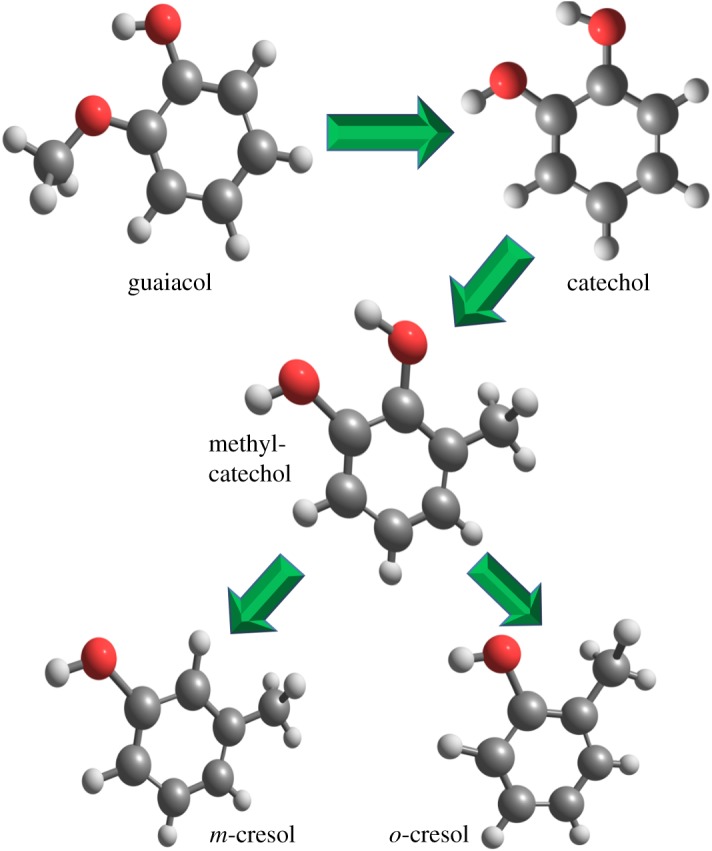
Reaction scheme of guaiacol conversion over Pt_3_ cluster.

In summary, a significant amount of work has been done on the HDO of guaiacol over different catalysts both experimentally and theoretically, producing various compounds such as catechol, phenol, benzene, cyclohexane, etc. A few experimental studies [24–26] have reported the formation of cresol from guaiacol over Re, Ni and Co catalysts. These experimental studies [24–26] support the formation of a cresol component from a guaiacyl lignin-derived model compound but no theoretical results are present in the open literature. Therefore, the present study aimed to produce cresol from guaiacol over a platinum catalyst. Cresol possesses three isomers, *o*-, *m*- and *p*-cresol, but this study focuses only on *o*- and *m*-cresol because experimental analyses by Yong & Yukihiko [26] do not support the formation of *p*-cresol. Both isomers are, in general, serving as important precursors in the production of industrial compounds as well as solvents in industry [27]. Further, we have carried out simulations for calculating the adsorption energies of guaiacol over 11 different catalysts such as Pt, Pd, Ru, Rh, Cu, Ni, Mo, Co, Ti, Fe and Zr. All catalysts are clusters of three atoms, for instance Pd_3_. The corresponding kinetic analyses have been carried out at a wide temperature range of 473–673 K at an interval temperature of 50 K and fixed pressure of 1 atm. The reaction scheme is shown in figure 1, in which guaiacol undergoes conversion to catechol, methylcatechol, and *o*- and *m*-cresol.

The size of the catalyst cluster has always been a matter of debate; however, to adsorb the guaiacol component, a large array of metals would be needed and a supported metal catalyst may also be reasonably predicted [28]. In the recent past, several computational works have been performed over small catalyst clusters [29,30]. For instance, Carneiro & Cruz [29] carried out DFT computations for formaldehyde adsorption over Pd_4_ catalysts (planar and tetrahedral) and validated their results with the literature counterparts. They reported that planar configuration of Pd_4_ provided high adsorption energy (highly negative) compared to tetrahedral Pd_4_; however, the complex of adsorbate and tetrahedral Pd_4_ was more stable according to absolute energy and gave the adsorption energy close to experimental values. It can be seen that the configurations of the catalyst cluster play a vital role in the prediction of accurate energetics; however, the Pt_3_ catalyst cluster cannot have different configurations unlike the four atoms models, i.e. planar and tetrahedral configurations of Pd_4_ catalyst adopted by Carneiro & Cruz [29]. Therefore, the present planar Pt_3_ configuration is a stable configuration. Various theoretical studies have also been reported in the literature for the three metal atoms catalyst model. For instance, Zhong *et al*. [30] carried out a methanol dehydrogenation study over Pt_3_ and PtAu_2_ catalyst clusters and concluded that the required activation energy was much lower with Pt_3_ catalyst compared to PtAu_2_; however, PtAu_2_ catalyst offered complete methanol dehydrogenation. Further, they compared their reaction progresses with the literature and found good agreement with that of bulk catalyst. Therefore, it can be seen that such small catalyst clusters could provide competitive energy parameters. Since in this work we have only studied the interaction of oxy-functional groups attached to a phenyl group with cluster atoms, the Pt_3_ cluster can be used for an initial insight. Further, the aromatic ring saturation is beyond the scope of this study as it might have required a bulk catalyst. Further, the noble metals perform better in the bio-oil upgrading process compared to other catalytic systems [2], therefore we have modelled our reaction schemes (shown in figure 1) over a Pt_3_ catalyst cluster.

## 2. Computational method

In the recent past, the DFT tool has emerged as one of the best computational tools in chemistry. It is advantageous compared to other contemporary theories because of its dependence on electron density, which is measurable and observable unlike the wave function theories. The wave function theories are the function of 4*N* variables, where *N* is number of electrons, whereas electron density is the function of only three spatial parameters no matter how complex the molecule is. Therefore, all minima and transition state structures have been optimized under the DFT [31,32] framework using a well-known hybrid–GGA functional, B3LYP (Becke, 3-parameter, Lee–Yang–Parr) [33,34] with the basis set of LANL2DZ [35], to consider the strong relativistic effects of Pt. The basis set 6–311+g(d,p) has been applied for C, H and O atoms. The stationary states of the potential energy surface (PES) have been affirmed by running a normal mode vibrational frequency calculation at the same level of theory as of the optimization calculation. One imaginary frequency has been found in each transition state structure which confirmed the true nature of the first-order saddle point on the PES and zero imaginary frequency has been found in all minimum structures. An intrinsic reaction coordinate (IRC) [36] analysis has been carried out at each transition state structure to link the transition state structure to the actual minima in both directions using the same theory as optimization theory. All minima from the IRC calculation are then subjected to normal mode vibrational frequency calculations to affirm the structures as true minima; zero imaginary frequency in each frequency result confirmed the structures as the true minima. All quantum calculations are carried out using Gaussian 09 [37] and Gauss View 5 [38] commercial software packages.

The Pt_3_ catalyst cluster [30] has been checked for its ground state configuration by running wave function stability analysis and the ground state laid in singlet spin state. Similarly, the complex of Pt_3_ and guaiacol has been checked for its ground state and it also laid in the singlet state. The adsorption energy [11,17], *E*_ads_, has been calculated by the following equation:

2.1 $$E_{\mathrm{ads}}=E_{\mathrm{complex}}-(E_{\mathrm{cluster}}+E_{\mathrm{substrate}}).$$

The upgrading processes occur at high temperatures; therefore, to obtain an insight into high temperature thermodynamics, the thermochemistry calculations [39,40] have also been performed at fixed pressure of 1 atm and at a wide temperature range of 473–673 K at an interval temperature of 50 K.

The reaction rate constant calculations have been carried out by employing Eyring's transition state theory [41]:

2.2 $$k(T)=\frac{k_{B}T}{h}\;\exp\;\left(\frac{-\Delta^{‡}G}{RT}\right),$$

where *k*_B_ is Boltzmann's constant (1.30662 × 10^−23^ J K^−1^) and *h* is Planck's constant (6.0626176 ×10^−34 ^J s^−1^). In this study, temperature (*T*) is varying for each calculation and *R* is the universal gas constant (1.987 cal/(mol K^−1^)).

## 3. Results and discussion

### 3.1. Adsorption energy

The adsorption energies obtained by several catalysts (shown in the electronic supplementary material, figure S1) are discussed followed by the potential energy surfaces of ‘guaiacol to catechol’, ‘guaiacol to methylcatechol (MC)’, ‘MC to *m*-cresol’ and ‘MC to *o*-cresol’ reactions. The adsorption energies (kcal/mol) are presented in table 1. All molecular structures are accompanied along with the PES figures; however, for better readability, they are also shown in the electronic supplementary material, figures S2–S6. In addition, the Cartesian coordinates of ‘guaiacol to catechol’ reaction are presented in the electronic supplementary material, table S2.

**Table 1.** The adsorption energy (*E*_ads_) of guaiacol over various catalysts. All energies are added with zero point vibrational energy (ZPVE).

catalyst	*E*_ads_ (kcal mol^−1^)
Zr_3_	−14.98
Pd_3_	−15.78
Ru_3_	−17.06
Mo_3_	−17.95
Ti_3_	−18.61
Pt_3_	−19.03
Cu_3_	−19.09
Ni_3_	−19.11
Co_3_	−20.09
Rh_3_	−22.51
Fe_3_	−22.92

The guaiacol component is adsorbed through on-top site configuration with one atom of the catalyst cluster. The oxygen atom of the methoxy group interacts with the cluster because the main aim of this study is the cleavage of the methyl group from the guaiacol component. As presented in table 1 the adsorption of guaiacol over a Zr_3_ catalyst surface has been calculated as the least exothermic adsorption, whereas guaiacol over a Fe_3_ catalyst surface possesses maximum exothermicity among all considered metals. However, the Pt_3_ cluster has been employed in this work because the noble metal catalyst performs better in HDO of the bio-oil model compound [2]. Another argument for choosing Pt catalyst among other noble metals is that the ‘guaiacol to catechol’ reaction has already been studied by Gao *et al*. [20] over Pt catalyst, therefore authors can use this reaction for validation and subsequently extend the present model for the rest of the reactions. Guaiacol adsorbs over the Pt_3_ catalyst cluster via on-top site configuration through the interaction of the oxygen atom of the methoxy group of guaiacol with an adsorption energy of −19.03 kcal mol^−1^. The distance between two binding atoms, i.e. Pt and O atoms, has been calculated as 2.22 Å and it is determined as the stable configuration to carry forward the reaction that forms the catechol compound.

### 3.2. Guaiacol to catechol and methylcatechol

**Figure 2.**
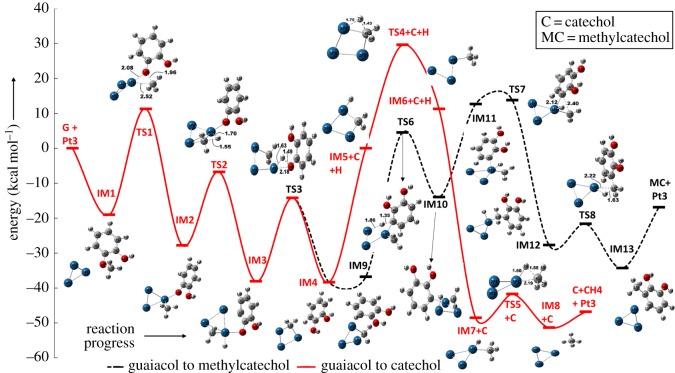
Potential energy profile of guaiacol HDO yielding catechol and methylcatechol. All energies are in kcal mol^−1^ with the addition of zero point vibrational energy (ZPVE) correction.

The formation of catechol component from guaiacol occurs through methyl group cleavage followed by the association of a hydrogen atom to the hydrogen–catecholate species. The PES starts from guaiacol and Pt_3_ catalyst cluster as individual species (figure 2) possessing the energy of 0 kcal mol^−1^ with respect to their own energies followed by the formation of IM1 (adsorbed guaiacol over Pt_3_). The intermediate IM1 proceeds to form IM2 with the energy barrier of 30.32 kcal mol^−1^. The IM2 structure depicts the cleaved methyl group from the guaiacol molecule showing a relative energy of −27.80 kcal mol^−1^ with respect to the G + Pt_3_. The demethylation reaction of guaiacol is studied by Verma & Kishore [9] in the absence of catalyst and they reported the required bond dissociation energy for methyl cleavage as 48.02 kcal mol^−1^. Figure 2 shows that the formation of catechol from guaiacol requires three transition states, namely TS1, TS2 and TS3, with the barrier heights as 30.32, 21.03 and 23.91 kcal mol^−1^, respectively; however, the methane molecule production from the methylene group after extraction of the catechol molecule requires two more elementary reaction steps with transition states TS4 and TS5. Clearly, it can be seen that among all energy barriers upto IM4 in the guaiacol to catechol reaction, the initial O─C bond scission appears as the rate determining step. The rate determining step requires 30.32 kcal mol^−1^ of energy, and thus this value is the activation energy, which closely matches with the value of Gao *et al*. [20]. They have developed the kinetic parameters experimentally, and based on ln *k* versus 1/*T* plot they reported the activation energy as 29.97 kcal mol^−1^ over a Pt/C catalyst surface. It can be seen that the difference between activation energies of the present study and that of Gao *et al*. [20] is only 0.35 kcal mol^−1^. The imaginary frequencies corresponding to TS1, TS2 and TS3 structures are calculated to be 502.03*i*, 901.88*i* and 1018.25*i* cm^−1^, respectively. Here ‘*i*’ corresponds to imaginary frequency and because all transition state structures have single imaginary frequencies, it affirms the fact that these transition states are first-order saddle points on the PES.

Further, IM2 structure progresses to form IM3. This elementary step shows the abstraction of hydrogen from the adsorbed methyl group. The cleaved hydrogen adsorbs on the same Pt atom on which hydrogen catecholate is already adsorbed. The transition state structure between IM2 and IM3 is shown in figure 2 as TS2, in which the bond breakage of H from C is clearly visible. The O─H association for the catechol formation from IM3 is depicted in the IM4 structure of figure 2. The transition state located for this reaction shows the sigma bond formation between O and H atoms, and at the same time the O atom disengages itself from the Pt atom. All three elementary reactions up to IM4 are calculated as exothermic reactions and IM4 is the point in the potential energy profile at which the catechol is formed. To move forward and in order to produce a methane molecule from the methylene group, two hydrogen atoms have been adsorbed in series. One hydrogen atom has been adsorbed over the Pt_3_ cluster with the methylene group in order to build the IM5 structure. The structure IM5 possesses zero relative energy in the potential energy profile (figure 2). On the other hand, IM7 shows −48.58 kcal mol^−1^ relative energy with respect to IM5 because of the adsorption of a hydrogen atom over the Pt_3_ cluster with the methyl group in order to produce a methane molecule. Two activated complexes after the catechol formation are expressed as TS4 and TS5, having imaginary frequencies of 968.34*i* and 813.04*i* cm^−1^, respectively. The fourth elementary step of the methyl formation is an endothermic reaction and it shows the energy barrier of 29.64 kcal mol^−1^, whereas the fifth elementary step is an exothermic reaction and shows a 6.85 kcal mol^−1^ energy barrier. The product complex of Pt_3_ and methane molecule shows 4.51 kcal mol^−1^ less energy compared to the summed energy of the individual Pt_3_ cluster, methane and catechol.

In order to form methylcatechol, the IM4 structure has been re-minimized with the interaction of hydrogen of the aromatic carbon of the catechol component with the Pt atom of the methylene adsorbed Pt_3_ cluster (see structure IM9 in figure 2 and electronic supplementary material, figure S2). Owing to this different configuration of adsorption, the energy corresponding to the complex of methylene adsorbed Pt_3_ cluster and catechol possesses −36.77 kcal mol^−1^ with respect to G + Pt_3_. The intermediates and transition state structures involved in the guaiacol to methylcatechol reaction are also shown in the electronic supplementary material, figures S2 and S3, respectively. Relative energetics of the structures is shown in the potential energy profile in figure 2 by the black dashed line. However, it should be noted that the intermediates upto IM4 and transition state structures upto TS3 for ‘guaiacol to catechol’ and ‘guaiacol to methylcatechol’ reactions are the same.

IM9 depicts the adsorption of catechol over the methylene adsorbed Pt_3_ cluster. Here the Pt atom interacts with hydrogen attached to aromatic carbon. For C─H bond scission, it is a necessary configuration. To cleave the C_aromatic_–H bond, a transition state structure has been located as TS6, having an energy barrier of 41.30 kcal mol^−1^. The single imaginary frequency associated with TS6 is calculated to be 436.34*i* cm^−1^. Two other transition states are located as TS7 (181.80*i *cm^−1^) and TS8 (792.2*i* cm^−1^) in order to produce the methylcatechol. The transition state structure TS7 connects its both minima as IM11 and IM12 through a minimum energy path, whereas TS8 links to IM12 and IM13. The respective energy barriers for these two elementary step reactions are reported as 1.11 and 6.06 kcal mol^−1^. The formation of IM12 from IM11 shows high exothermicity compared to the IM12 → IM13 elementary step reaction. The stationary point IM13 on PES shows the formation of methylcatechol over Pt_3_ as the product complex. The desorption of methylcatechol from the Pt_3_ cluster requires 4.52 kcal mol^−1^ of energy. The individual species Pt_3_ and methylcatechol possess relative energy of −51.35 kcal mol^−1^ and the product complex depicts energy of −46.83 kcal mol^−1^ relative to G + Pt_3_. It is clear that the elementary step which involves C─H bond scission appears as the rate determining step and thus the activation energy of guaiacol to methylcatechol reaction is calculated as 41.3 kcal mol^−1^. The methylcatechol produced from the guaiacol HDO reaction acts as the reactant for the formation of *o*- and *m*-cresol, discussed in the following subsections.

### 3.3. Methylcatechol to cresol

**Figure 3.**
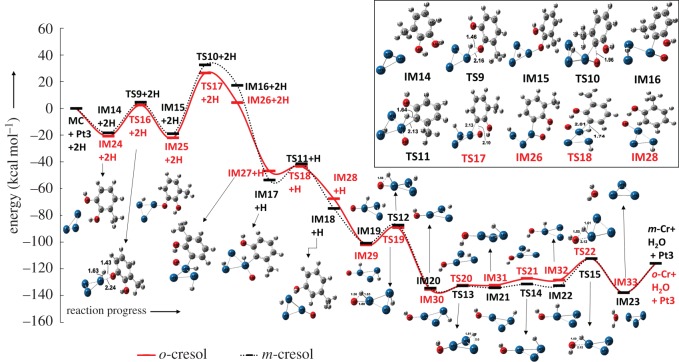
Potential energy profile of methylcatechol (MC) HDO reactions yielding *o*-cresol (*o*-Cr) and *m*-cresol (*m*-Cr). All energies are in kcal mol^−1^ with the addition of zero point vibrational energy (ZPVE) correction. The structures from IM19 to IM23 and IM29 to IM33 carry the energetics of *m*-cresol and *o*-cresol, respectively.

Methylcatechol is hydrodeoxygenated into two important compounds, *o*-cresol and *m*-cresol. Both product formations from methylcatechol are discussed separately and the potential energy profiles can be seen in figure 3.

#### 3.3.1. Methylcatechol to *m*-cresol

The potential energy profile starts from the summed energetics of individual methylcatechol, 2H and Pt_3_ cluster species showing zero relative energy as reference for other structures. The intermediate IM14 is adsorbed methylcatechol over Pt_3_ catalyst and it is worth noting that structures in potential energy profile (figure 3) in between IM14 and IM16 carry the energy of two hydrogen atoms, whereas the structures in between IM17 and IM18 carry the energy of one hydrogen atom. In the following discussions, structures are denoted as IMn or TSn without H and 2H. The methylcatechol binds to the Pt_3_ cluster with the central hydroxyl group and the adsorption energy is predicted as −18.38 kcal mol^−1^. In the IM14 structure, the oxygen atom interacts with one Pt atom and progresses to form IM15 with a transition state structure TS9 (1100.72*i* cm^−1^). The energy barrier for this elementary step is calculated as 23.01 kcal mol^−1^ with a little exothermicity. Intermediate IM15 now progresses to the C─O bond scission in order to detach the oxygen atom from the substrate. This process occurs through TS10 (420.02*i* cm^−1^), showing an energy barrier of 51.52 kcal mol^−1^ in the potential energy profile. Further, an additional hydrogen atom adsorption on the cluster, consequently forming a different intermediate, IM17, which shows −53.66 kcal mol^−1^ relative energy to the reference energy. The newly adsorbed hydrogen interacts with 3-methyl-hydroxyphenyl in order to produce *m*-cresol and this reaction occurs through a transition state TS11, which possesses relative energy of −41.53 kcal mol^−1^. Single imaginary frequency for TS11 is calculated as 797.29*i* cm^−1^ and, unlike the second elementary step, this step does show exothermic nature. IM18 demonstrates the binding of individual species *m*-cresol, O and H on the Pt_3_ cluster. The extraction of *m*-cresol from IM18 and adsorption of an additional hydrogen atom displays a stationary state on PES as IM19, and it is to be noted that structures from IM19 to IM23 carry *m*-cresol's energetics in the potential energy profile (figure 3). As shown in figure 3, it is observed that the reaction progresses through IM19 → TS12 → IM20 → TS13 → IM21 → TS14 → IM22 → TS15 → IM23 → *m*-cresol + H_2_O + Pt_3_. These elementary reaction steps from IM19 are to form water compound on the Pt_3_ cluster. The single imaginary frequency corresponding to transition state structures, i.e. TS12, TS13, TS14 and TS15, after IM19 are calculated to be 1190.76*i*, 395.99*i*, 378.55*i* and 1035.44*i* cm^−1^, respectively, and corresponding energy barriers are calculated as 13.51 kcal mol^−1^, 1.74 kcal mol^−1^, 2.91 kcal mol^−1^ and 20.32 kcal mol^−1^, respectively. It can be seen clearly that the C─O bond scission reaction step of methylcatechol to *m*-cresol reaction is the rate determining step, thus the activation energy for the methylcatechol to *m*-cresol reaction is 51.52 kcal mol^−1^. The desorption of water compound from the Pt_3_ cluster demands 22.02 kcal mol^−1^ of energy as the individual water, Pt_3_ cluster and *m*-cresol species possess 22.02 kcal mol^−1^ higher energy compared to their complex.

#### 3.3.2. Methylcatechol to *o*-cresol

In this section, *o*-cresol formation from the methylcatechol over Pt_3_ cluster is discussed. The potential energy profile of *o*-cresol formation is shown in figure 3. As noted for *m*-cresol about the notations in PES, here too the structures in the potential energy profile (figure 3) in between IM24 and IM26 carry the energy of two hydrogen atoms and the structures in between IM27 and IM28 carry the energy of one hydrogen atom. Here too the structures are denoted as IMn or TSn without H and 2H.

The adsorption energy of methylcatechol over Pt_3_ cluster is calculated as −20.85 kcal mol^−1^. It can be seen that the IM24 configuration for the formation of *o*-cresol is more stable than IM14. It should be noted that the interacting hydroxyl group of methylcatechol with cluster for *o*-cresol production is different than the case of *m*-cresol. The reaction progresses as per the *m*-cresol reaction but with slight energy changes, for instance, O─H bond scission for *o*-cresol formation occurs with an energy barrier of 23.3 kcal mol^−1^, whereas the same process required 23.01 kcal mol^−1^ in the *m*-cresol reaction profile. Thermochemistry suggests that O─H bond scission is more exothermic for *o*-cresol formation compared with *m*-cresol. The intermediate structure IM25 demonstrates the cleaved O─H bond over cluster, however the cleaved hydrogen atom and the rest of the molecule are adsorbed on different Pt atoms of the cluster. The IM25 further progresses to the C─O bond cleavage which requires an energy barrier of 48.5 kcal mol^−1^, whereas C─O bond cleavage in the *m*-cresol potential energy profile requires 51.52 kcal mol^−1^. Similar to the C─O bond scission elementary step of the *m*-cresol reaction, this is also an endothermic reaction step. The transition state structures for both elementary steps possess an imaginary frequency of 1102.94*i* and 357.06*i* cm^−1^, respectively. The C─O bond cleavage is followed by an additional hydrogen atom adsorption over a Pt atom of the cluster which is a minimum structure on PES, shown as IM27 in figure 3 and electronic supplementary material, figure S6, with relative energy of −46.82 kcal mol^−1^. The additional hydrogen atom interacts with the carbon atom attached to the cluster in order to produce the *o*-cresol. The product complex and its energetics are shown in figure 3. The transition state located in between the IM27 and IM28 possesses an imaginary frequency of 708.75*i* cm^−1^ and this first-order saddle point on PES shows a relative energy of −43.44 kcal mol^−1^, which is slightly less compared to TS11 of the *m*-cresol reaction. The reaction follows similarly to the *m*-cresol reaction. The extraction of *o*-cresol and adsorption of an additional hydrogen atom on the rest of the substrate over the cluster forms an intermediate which is shown as IM29 in figure 3. The reaction progress after IM29 follows the reaction steps IM29 → TS19 → IM30 → TS20 → IM31 → TS21 → IM32 → TS22 → IM33. The transition states TS19, TS20, TS21 and TS22 possess imaginary frequencies of 1188.25*i*, 402.92*i*, 445.07*i* and 1035.51*i *cm^−1^, respectively. The energy barriers for these elementary steps, i.e. IM29 → IM30, IM30 → IM31, IM31 → IM32 and IM32 → IM33, are calculated as 12.8, 3.0, 5.02 and 16.08 kcal mol^−1^, respectively. Here too, the C─O bond scission appears as the rate determining step amongst all reaction steps of the methylcatechol to *o*-cresol reaction. Thus, the activation energy of methylcatechol to *o*-cresol reaction is obtained as 48.5 kcal mol^−1^. Similar to the case of *m*-cresol, desorption of water from the cluster requires 22.02 kcal mol^−1^ in the case of *o*-cresol.

### 3.4. Kinetic modelling

**Figure 4.**
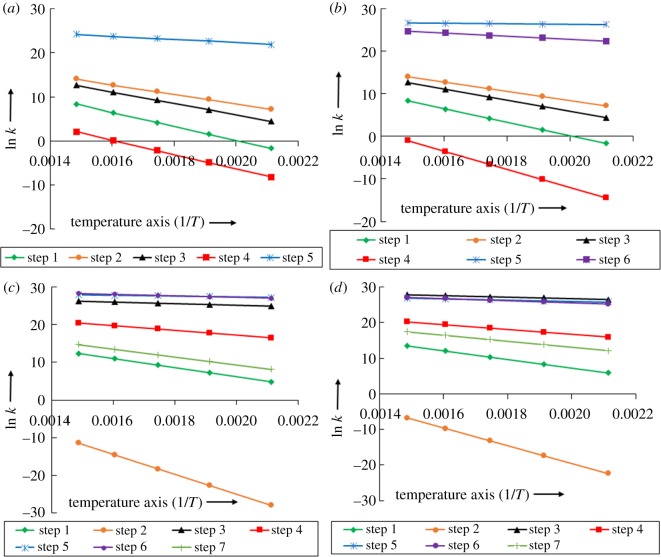
ln *k* versus 1/*T* trends of all reactions participating in the conversion of guaiacol into catechol (*a*), methylcatechol (*b*), *m*-cresol (*c*) and *o*-cresol (*d*) in the presence of Pt_3_ catalyst cluster.

The kinetic parameters of any reaction are the key findings and they contribute in the designing of a reaction model in any reactor. For instance, if one opts to optimize the flow behaviour of any reacting molecule on a catalytic or non-catalytic system inside the reactor then one has to have the kinetics of that reaction, i.e. activation energy, rate constant, pre-exponential factor, etc. The kinetics of all discussed reactions, i.e. guaiacol to catechol, guaiacol to methylcatechol, methylcatechol to *m*-cresol and methylcatechol to *o*-cresol, are calculated using the thermochemistry obtained from the vibrational frequency analyses. The temperature analyses are carried out at five different temperatures, i.e. 473, 523, 573, 623 and 673 K (table 2). The Arrhenius equations have been proposed for each elementary step of each particular reaction in the electronic supplementary material, table S1. The plots of ln *k* versus 1/*T* for all elementary steps of particular reactions are shown in figure 4.

**Table 2.** The reaction rate constants of every elementary step of each particular reaction at different temperatures.

		*k* (s^−1^)				
elementary steps	*E*_a_ (kcal mol^−1^)	473 (K)	523 (K)	573 (K)	623 (K)	673 (K)
guaiacol to catechol reaction						
TS1	30.32	0.19	4.80	68.16	585.44	4286.31
TS2	21.03	1312.86	11 795.4	72 770	309 870	1 249 017
TS3	23.91	84.99	1170.49	10319.5	59510.2	310 975
TS4	29.64	0.00028	0.00777	0.11962	1.0773	8.08273
TS5	6.85	3.2 × 10^9^	6.9 × 10^9^	1.3 × 10^10^	2 × 10^10^	3.4 × 10^10^
guaiacol to methylcatechol reaction						
TS1–TS3	are same as guaiacol to catechol reaction					
TS6	41.3	5.3 × 10^−7^	4 × 10^−5^	0.00141	0.02851	0.36967
TS7	1.11	2.6 × 10^11^	3 × 10^11^	3.3 × 10^11^	3.6 × 10^11^	3.9 × 10^11^
TS8	6.66	5.4 × 10^9^	1.1 × 10^10^	2.1 × 10^10^	3.5 × 10^10^	5.5 × 10^10^
methylcatechol to *m*-cresol reaction						
TS9	23.01	128.14	1441.59	10 690	57816.4	244 215
TS10	51.52	6.8 × 10^−13^	1.4 × 10^−10^	1.1 × 10^−8^	4.6 × 10^−7^	1.1 × 10^−5^
TS11	12.13	6.9 × 10^10^	1.1 × 10^11^	1.5 × 10^11^	2 × 10^11^	2.5 × 10^11^
TS12	13.51	1.5 × 10^7^	5.4 × 10^7^	1.5 × 10^8^	3.7 × 10^8^	7.9 × 10^8^
TS13	1.74	7.2 × 10^11^	8.9 × 10^11^	1.1 × 10^12^	1.2 × 10^12^	1.4 × 10^12^
TS14	2.91	5.9 × 10^11^	8.6 × 10^11^	1.2 × 10^12^	1.5 × 10^12^	1.9 × 10^12^
TS15	20.32	3345.48	27870.6	160 662	699 794	2 451 438
methylcatechol to *o*-cresol reaction						
TS16	23.3	356.62	4122.35	31261.7	172 026	737 407
TS17	48.5	1.9 × 10^−10^	2.9 × 10^−8^	1.9 × 10^−6^	6.3 × 10^−5^	0.00124
TS18	3.38	3.3 × 10^11^	5.1 × 10^11^	7.2 × 10^11^	9.7 × 10^11^	1.2 × 10^12^
TS19	12.8	8 831 776	3.4 × 10^7^	1.1 × 10^8^	2.7 × 10^8^	6.1 × 10^8^
TS20	3.2	1.6 × 10^11^	2.2 × 10^11^	3 × 10^11^	3.9 × 10^11^	4.8 × 10^11^
TS21	5.02	9.4 × 10^10^	1.7 × 10^11^	2.8 × 10^11^	4.2 × 10^11^	5.9 × 10^11^
TS22	16.08	195 662	1 055 828	4 250 431	1.4 × 10^7^	3.7 × 10^7^

As discussed earlier, the guaiacol to catechol reaction requires five elementary steps; therefore, five straight lines corresponding to each elementary step are shown with five points of temperatures in figure 4*a*. It can be seen in figure 4*a* that the slope of step 1 is highly negative among all five and, therefore, it possesses the highest activation energy requirement. The rate constant improves as the temperature increases and out of all the discussed temperatures, 673 K performs best. Gao *et al*. [20] showed the ln *k* versus 1/*T* plot for five reactions in which the guaiacol to catechol reaction has been explained as a second order of reaction kinetics experimentally. The guaiacol to methylcatechol reaction has been shown in six elementary steps and the highest activation energy requirement is from the fourth elementary step, i.e. C─H bond scission (figure 4*b*). Here too, the temperature affects rate constant proportionally, i.e. the rate constant increases with increasing temperature. The ln *k* versus 1/*T* plot for this reaction indicates all elementary steps as straight lines. On the other hand, methylcatechol to *m*-cresol reaction is explained in seven elementary reaction steps in figure 4*c*. As discussed earlier, the second elementary step of methylcatechol to *m*-cresol, i.e. C─O bond scission, is the rate determining step, and the same can be seen in figure 4*c* because the second step is a highly negative sloping straight line. Similar phenomena can be seen for the methylcatechol to *o*-cresol reaction also (figure 4*d*). The rate constant increases with increasing temperature and both ln *k* versus 1/*T* plots of *m*- and *o*-cresol show straight lines with negative slopes.

It can be observed that the formation of catechol from demethylation of guaiacol over the present cluster model required an activation energy of 30.32 kcal mol^−1^, which is very close to the reported activation energy value found by to Gao *et al*. [20] using experimental kinetic analysis. Therefore, the present small cluster model has the potential for studying the functional-based cleavage reactions of phenyl containing compounds; however, its performance cannot be entirely concluded without having a proper and significant amount of experimental evidence. On the other hand, the production of cresol from guaiacol using computations is completely novel work and no one as far as the authors' knowledge has tried this; a few experimental works [24–26] over different catalysts do suggest its production but those studies do not report elementary reaction mechanisms. In addition, most experimental analyses undergo the study of selectivity, yield, catalytic activity, etc., therefore the validation of formation of cresols cannot be done at this stage.

Furthermore, recent works [29,30] based on such small catalyst clusters have proved their catalytic performances compared to their experimental or theoretical counterparts, but these works were carried out for different components. Therefore, the reliability of the Pt_3_ catalyst model for the present kind of study may require its testing by experimental kinetic analysis of HDO of guaiacol which accompanies the productions of cresols along with other hydrodeoxygenated products. Nevertheless, the present model of Pt_3_ catalyst cluster for this work has been considered to deliver a preliminary reaction scheme for the conversion mechanism of guaiacol to cresol and the kinetics could be used as first-order approximation in the absence of experimental results.

## 4. Conclusion

The guaiacol conversion process yielding catechol, 3-methylcatechol, *m*-cresol and *o*-cresol has been studied under the DFT framework. The key conclusions are as follows.

The adsorption energy of guaiacol on Pt_3_ cluster indicates exothermic adsorption. The use of the Pt_3_ cluster can be very insightful in order to have an initial picture of any particular reaction because the activation energy of the ‘guaiacol to catechol’ reaction is in good approximation with the literature. The O─H bond scission has been calculated as the rate determining step for guaiacol to catechol reaction, whereas C─H bond scission of the phenyl ring of catechol appears as the rate determining step for guaiacol to methylcatechol reaction. The 3-methylcatechol to *m*- and *o*-cresol reactions show the C─O bond cleavage as the rate determining step. Increasing temperature increases the rate constants of all reactions.

## Supplementary Material

Electronic Supplementary Material for the manuscript entitled “Platinum Catalyzed Hydrodeoxygenation of Guaiacol in Illumination of Cresol Production: A DFT Study”.

## Data accessibility

The datasets supporting this article have been uploaded as part of the electronic supplementary material.
