# Supplementary material for: Platinum catalyzed hydrodeoxygenation of guaiacol in illumination of cresol production: a density functional theory study
Source: R Soc Open Sci. 2017 Nov 8;4(11):170650. doi: 10.1098/rsos.170650 (PMC5717632; doi:10.1098/rsos.170650)
Supplement: Electronic Supplementary Material for the manuscript entitled “Platinum Catalyzed Hydrodeoxygenation of Guaiacol in Illumination of Cresol Production: A DFT Study”. [file rsos170650supp1.docx]

**Table S1.** The Arrhenius equations of each elementary reaction step in the conversion process of guaiacol into catechol, methylcatechol, *m*-, and *o*-cresol over Pt_3_ catalyst cluster. These Arrhenius relations are reported for the temperature range of 473-673 K at an interval temperature of 50 K.

| **Elementary Steps** | **E_a_ (kcal/mol)** | **Arrhenius Equation** |
| --- | --- | --- |
| **Guaiacol to Catechol Reaction** | | |
| TS1 | 30.32 | $k= 5.86 \times10^{13} \exp(-15752/T)$ |
| TS2 | 21.03 | $k= 1.09 \times10^{13} \exp(-10793/T)$ |
| TS3 | 23.91 | $k= 8.85 \times10^{12} \exp(-11788/T)$ |
| TS4 | 29.64 | $k= 4.23\times10^{11} \exp(-15575/T)$ |
| TS5 | 6.85 | $k= 7.86 \times10^{12} \exp(-3689.5/T)$ |
| **Guaiacol to Methylcatechol Reaction** | | |
| TS1- TS3 | Are same as guaiacol to catechol reaction | |
| TS6 | 41.3 | $k= 1.84 \times10^{13} \exp(-21255/T)$ |
| TS7 | 1.11 | $k= 9.6 \times10^{11} \exp(-608.77/T)$ |
| TS8 | 6.66 | $k= 1.30 \times10^{13} \exp(-3682.2/T)$ |
| **Methylcatechol to *m*-cresol Reaction** | | |
| TS9 | 23.01 | $k= 1.17 \times10^{13} \exp(-11923/T)$ |
| TS10 | 51.52 | $k= 9.05 \times10^{11} \exp(-26257/T)$ |
| TS11 | 12.13 | $k= 5.57 \times10^{12} \exp(-2076.3/T)$ |
| TS12 | 13.51 | $k= 8.06 \times10^{12} \exp(-6223.1/T)$ |
| TS13 | 1.74 | $k= 6.43 \times10^{12} \exp(-1033.4/T)$ |
| TS14 | 2.91 | $k= 2.95 \times10^{13} \exp(-1841.1/T)$ |
| TS15 | 20.32 | $k= 1.40\times10^{13} \exp(-10475/T)$ |
| **Methylcatechol to *o*-cresol Reaction** | | |
| TS16 | 23.3 | $k= 4.39 \times10^{13} \exp(-12066/T)$ |
| TS17 | 48.5 | $k= 1.36 \times10^{13} \exp(-24878/T)$ |
| TS18 | 3.38 | $k= 2.57 \times10^{13} \exp(-2046.5/T)$ |
| TS19 | 12.8 | $k= 1.27 \times10^{13} \exp(-6699.2/T)$ |
| TS20 | 3.2 | $k= 6.89 \times10^{12} \exp(-1789.7/T)$ |
| TS21 | 5.02 | $k= 4.22 \times10^{13} \exp(-2881.3/T)$ |
| TS22 | 16.08 | $k= 8.65\times10^{12} \exp(-8322.5/T)$ |

**Table S2.** The Cartesian coordinates of all involved molecular structures in ‘Guaiacol to catechol’ reaction scheme.

| **S. No.** | **Structure** | **xyz-coordinates** |
| --- | --- | --- |
|  | IM1 | \| Tag \| Symbol \| X \| Y \| Z \| \| --- \| --- \| --- \| --- \| --- \| \| 1 \| Pt \| 0.174374 \| -0.67364 \| -0.09138 \| \| 2 \| Pt \| -2.32568 \| -0.69137 \| -0.42104 \| \| 3 \| Pt \| -1.21228 \| 1.377199 \| 0.469588 \| \| 4 \| C \| 3.364934 \| -0.08407 \| -0.0479 \| \| 5 \| C \| 4.704869 \| -0.46262 \| 0.187176 \| \| 6 \| C \| 5.745114 \| 0.248479 \| -0.43937 \| \| 7 \| C \| 5.450267 \| 1.3015 \| -1.32358 \| \| 8 \| C \| 4.111354 \| 1.647365 \| -1.58546 \| \| 9 \| C \| 3.0706 \| 0.955882 \| -0.93987 \| \| 10 \| H \| 6.780301 \| -0.03634 \| -0.2525 \| \| 11 \| H \| 6.260425 \| 1.836244 \| -1.81195 \| \| 12 \| H \| 3.873881 \| 2.452339 \| -2.27439 \| \| 13 \| H \| 2.025045 \| 1.203978 \| -1.11018 \| \| 14 \| O \| 4.950686 \| -1.54337 \| 1.04672 \| \| 15 \| H \| 5.908572 \| -1.79062 \| 1.049946 \| \| 16 \| O \| 2.285227 \| -0.76963 \| 0.577273 \| \| 17 \| C \| 2.329035 \| -0.85453 \| 2.074156 \| \| 18 \| H \| 2.270121 \| 0.163438 \| 2.474445 \| \| 19 \| H \| 1.446414 \| -1.4265 \| 2.353314 \| \| 20 \| H \| 3.250438 \| -1.36131 \| 2.369459 \| |
|  | TS1 | \| Tag \| Symbol \| X \| Y \| Z \| \| --- \| --- \| --- \| --- \| --- \| \| 1 \| Pt \| 0.221129 \| -0.66073 \| -0.04265 \| \| 2 \| Pt \| -2.29103 \| -0.67713 \| -0.44305 \| \| 3 \| Pt \| -1.20536 \| 1.380714 \| 0.45438 \| \| 4 \| C \| 3.318026 \| -0.18085 \| -0.22059 \| \| 5 \| C \| 4.622945 \| -0.49404 \| 0.240479 \| \| 6 \| C \| 5.725363 \| 0.259027 \| -0.20266 \| \| 7 \| C \| 5.535649 \| 1.334062 \| -1.09124 \| \| 8 \| C \| 4.239914 \| 1.661199 \| -1.53596 \| \| 9 \| C \| 3.13792 \| 0.907174 \| -1.09784 \| \| 10 \| H \| 6.726465 \| 0.007911 \| 0.146405 \| \| 11 \| H \| 6.393109 \| 1.910393 \| -1.42817 \| \| 12 \| H \| 4.088237 \| 2.492221 \| -2.21898 \| \| 13 \| H \| 2.128955 \| 1.134378 \| -1.43262 \| \| 14 \| O \| 4.74706 \| -1.56228 \| 1.137438 \| \| 15 \| H \| 5.685402 \| -1.75875 \| 1.337163 \| \| 16 \| O \| 2.266688 \| -0.9941 \| 0.174648 \| \| 17 \| C \| 1.644608 \| -0.83821 \| 2.027821 \| \| 18 \| H \| 0.801192 \| -0.24244 \| 2.384524 \| \| 19 \| H \| 1.570242 \| -1.91075 \| 2.184764 \| \| 20 \| H \| 2.620279 \| -0.41491 \| 2.253508 \| |
|  | IM2 | \| Tag \| Symbol \| X \| Y \| Z \| \| --- \| --- \| --- \| --- \| --- \| \| 1 \| Pt \| 0.205607 \| -0.73627 \| -0.33216 \| \| 2 \| Pt \| -2.37086 \| -0.47412 \| -0.13054 \| \| 3 \| Pt \| -0.85455 \| 1.467347 \| 0.328652 \| \| 4 \| C \| 3.291264 \| -0.28499 \| -0.55174 \| \| 5 \| C \| 4.011503 \| -0.62624 \| 0.628624 \| \| 6 \| C \| 5.227615 \| 0.010524 \| 0.933912 \| \| 7 \| C \| 5.754804 \| 0.990192 \| 0.072028 \| \| 8 \| C \| 5.060186 \| 1.332215 \| -1.1059 \| \| 9 \| C \| 3.845522 \| 0.698134 \| -1.40881 \| \| 10 \| H \| 5.763364 \| -0.26135 \| 1.843422 \| \| 11 \| H \| 6.69573 \| 1.474853 \| 0.318074 \| \| 12 \| H \| 5.461888 \| 2.084342 \| -1.77953 \| \| 13 \| H \| 3.292777 \| 0.940169 \| -2.31258 \| \| 14 \| O \| 3.46515 \| -1.61283 \| 1.462317 \| \| 15 \| H \| 4.060271 \| -1.81678 \| 2.212713 \| \| 16 \| O \| 2.123959 \| -0.89342 \| -0.91466 \| \| 17 \| C \| 0.265404 \| -1.63729 \| 1.505198 \| \| 18 \| H \| -0.46016 \| -2.45324 \| 1.480778 \| \| 19 \| H \| 1.304073 \| -1.96641 \| 1.610948 \| \| 20 \| H \| -0.02448 \| -0.89016 \| 2.259959 \| |
|  | TS2 | \| Tag \| Symbol \| X \| Y \| Z \| \| --- \| --- \| --- \| --- \| --- \| \| 1 \| Pt \| 0.337981 \| -0.56874 \| -0.46978 \| \| 2 \| Pt \| -2.12043 \| -0.76644 \| 0.37572 \| \| 3 \| Pt \| -1.10017 \| 1.576219 \| -0.04766 \| \| 4 \| C \| 3.208822 \| 0.189484 \| -0.36614 \| \| 5 \| C \| 4.074429 \| -0.85602 \| 0.066333 \| \| 6 \| C \| 5.223246 \| -0.56172 \| 0.82086 \| \| 7 \| C \| 5.528329 \| 0.77273 \| 1.152535 \| \| 8 \| C \| 4.674689 \| 1.815716 \| 0.739767 \| \| 9 \| C \| 3.519688 \| 1.520633 \| -0.00271 \| \| 10 \| H \| 5.878572 \| -1.36876 \| 1.148035 \| \| 11 \| H \| 6.422611 \| 0.9917 \| 1.730141 \| \| 12 \| H \| 4.907664 \| 2.845907 \| 0.995176 \| \| 13 \| H \| 2.847423 \| 2.305174 \| -0.34015 \| \| 14 \| O \| 3.704163 \| -2.16212 \| -0.26998 \| \| 15 \| H \| 4.375974 \| -2.81299 \| 0.020675 \| \| 16 \| O \| 2.142834 \| -0.08429 \| -1.17917 \| \| 17 \| C \| -0.55635 \| -2.10259 \| 0.546429 \| \| 18 \| H \| -0.93771 \| -3.02263 \| 0.078985 \| \| 19 \| H \| 0.737469 \| -2.20803 \| -0.29979 \| \| 20 \| H \| -0.2007 \| -2.22936 \| 1.571522 \| |
|  | IM3 | \| Tag \| Symbol \| X \| Y \| Z \| \| --- \| --- \| --- \| --- \| --- \| \| 1 \| Pt \| -0.2273 \| -1.36966 \| -0.29224 \| \| 2 \| Pt \| -2.14877 \| 0.371586 \| 0.221982 \| \| 3 \| Pt \| 0.378186 \| 1.275967 \| -0.11023 \| \| 4 \| C \| 2.424291 \| -0.76732 \| -0.46486 \| \| 5 \| C \| 3.380369 \| -0.74784 \| 0.575293 \| \| 6 \| C \| 3.940405 \| 0.483237 \| 1.009168 \| \| 7 \| C \| 3.542314 \| 1.701297 \| 0.44928 \| \| 8 \| C \| 2.617345 \| 1.73653 \| -0.6375 \| \| 9 \| C \| 2.079025 \| 0.491352 \| -1.13967 \| \| 10 \| H \| 4.662945 \| 0.476888 \| 1.824924 \| \| 11 \| H \| 3.965225 \| 2.629823 \| 0.82288 \| \| 12 \| H \| 2.521745 \| 2.643023 \| -1.22984 \| \| 13 \| H \| 1.74365 \| 0.39649 \| -2.17232 \| \| 14 \| O \| 3.686756 \| -1.95751 \| 1.18905 \| \| 15 \| H \| 4.334464 \| -1.849 \| 1.916008 \| \| 16 \| O \| 1.7142 \| -1.88045 \| -0.79438 \| \| 17 \| C \| -1.44832 \| -1.16167 \| 1.305239 \| \| 18 \| H \| -2.18312 \| -1.95843 \| 1.465006 \| \| 19 \| H \| -0.7271 \| -2.85732 \| -0.49637 \| \| 20 \| H \| -0.90379 \| -0.86689 \| 2.208438 \| |
|  | TS3 | \| Tag \| Symbol \| X \| Y \| Z \| \| --- \| --- \| --- \| --- \| --- \| \| 1 \| Pt \| 0.415368 \| -1.05699 \| 0.018403 \| \| 2 \| Pt \| -1.39696 \| 1.630809 \| -0.1185 \| \| 3 \| Pt \| -2.10951 \| -0.79597 \| 0.038359 \| \| 4 \| C \| 3.561703 \| -0.53407 \| 0.046443 \| \| 5 \| C \| 3.81434 \| 0.584086 \| 0.88097 \| \| 6 \| C \| 4.890319 \| 1.446819 \| 0.620553 \| \| 7 \| C \| 5.731378 \| 1.198205 \| -0.48262 \| \| 8 \| C \| 5.485628 \| 0.093446 \| -1.32084 \| \| 9 \| C \| 4.399357 \| -0.76338 \| -1.0611 \| \| 10 \| H \| 5.076869 \| 2.299773 \| 1.272082 \| \| 11 \| H \| 6.568036 \| 1.862572 \| -0.68109 \| \| 12 \| H \| 6.132897 \| -0.10028 \| -2.17193 \| \| 13 \| H \| 4.193574 \| -1.62323 \| -1.69253 \| \| 14 \| O \| 2.917845 \| 0.753405 \| 1.947812 \| \| 15 \| H \| 3.121109 \| 1.537241 \| 2.496441 \| \| 16 \| O \| 2.542607 \| -1.42174 \| 0.354146 \| \| 17 \| C \| 0.330481 \| 0.857402 \| -0.68414 \| \| 18 \| H \| 1.171707 \| 1.41839 \| -0.24856 \| \| 19 \| H \| 1.498964 \| -0.77917 \| 1.210015 \| \| 20 \| H \| 0.37969 \| 0.763723 \| -1.77988 \| |
|  | IM4 | \| Tag \| Symbol \| X \| Y \| Z \| \| --- \| --- \| --- \| --- \| --- \| \| 1 \| Pt \| -0.41017 \| -0.9016 \| -0.22739 \| \| 2 \| Pt \| 1.595229 \| 1.582185 \| -0.04332 \| \| 3 \| Pt \| 2.089256 \| -0.89452 \| 0.195072 \| \| 4 \| C \| -3.62261 \| -0.33099 \| -0.03898 \| \| 5 \| C \| -4.60475 \| 0.400218 \| -0.73301 \| \| 6 \| C \| -5.66162 \| 1.000828 \| -0.0366 \| \| 7 \| C \| -5.72406 \| 0.858828 \| 1.365135 \| \| 8 \| C \| -4.73924 \| 0.123794 \| 2.051336 \| \| 9 \| C \| -3.67559 \| -0.47753 \| 1.347506 \| \| 10 \| H \| -6.42174 \| 1.56875 \| -0.5699 \| \| 11 \| H \| -6.53887 \| 1.32246 \| 1.913242 \| \| 12 \| H \| -4.7904 \| 0.019751 \| 3.131757 \| \| 13 \| H \| -2.8957 \| -1.0376 \| 1.853959 \| \| 14 \| O \| -4.40158 \| 0.450251 \| -2.12392 \| \| 15 \| H \| -5.06709 \| 0.979888 \| -2.6035 \| \| 16 \| O \| -2.57688 \| -0.90727 \| -0.79692 \| \| 17 \| C \| -0.26178 \| 1.014483 \| 0.379256 \| \| 18 \| H \| -0.98151 \| 1.639971 \| -0.18084 \| \| 19 \| H \| -2.71483 \| -0.72704 \| -1.75458 \| \| 20 \| H \| -0.42055 \| 1.039172 \| 1.468401 \| |
|  | IM5 | \| Tag \| Symbol \| X \| Y \| Z \| \| --- \| --- \| --- \| --- \| --- \| \| 1 \| Pt \| -1.51558 \| 0.373098 \| -0.09666 \| \| 2 \| Pt \| 1.226604 \| 0.949381 \| -0.0815 \| \| 3 \| Pt \| 0.360635 \| -1.48438 \| 0.047768 \| \| 4 \| C \| -0.31507 \| 1.468881 \| 1.072927 \| \| 5 \| H \| -0.54912 \| 2.54303 \| 0.988574 \| \| 6 \| H \| -0.25362 \| 1.092352 \| 2.098646 \| \| 7 \| H \| -2.89638 \| 0.179892 \| 0.645687 \| |
|  | TS4 | \| Tag \| Symbol \| X \| Y \| Z \| \| --- \| --- \| --- \| --- \| --- \| \| 1 \| Pt \| -1.58493 \| -0.13249 \| -0.00013 \| \| 2 \| Pt \| 0.9256 \| 1.222681 \| -0.00011 \| \| 3 \| Pt \| 0.811838 \| -1.30848 \| 0.000101 \| \| 4 \| C \| -1.16379 \| 1.810432 \| 0.00107 \| \| 5 \| H \| -1.19675 \| 2.410393 \| -0.91991 \| \| 6 \| H \| -1.19573 \| 2.409229 \| 0.922839 \| \| 7 \| H \| -2.52012 \| 1.344575 \| 0.001595 \| |
|  | IM6 | \| Tag \| Symbol \| X \| Y \| Z \| \| --- \| --- \| --- \| --- \| --- \| \| 1 \| Pt \| -1.33882 \| -0.61078 \| 0.000184 \| \| 2 \| Pt \| 0.263039 \| 1.366651 \| 0.000035 \| \| 3 \| Pt \| 1.462556 \| -0.77177 \| -0.0001 \| \| 4 \| C \| -3.23725 \| 0.106491 \| -0.00096 \| \| 5 \| H \| -3.43429 \| 0.700124 \| -0.90254 \| \| 6 \| H \| -3.43525 \| 0.700163 \| 0.900405 \| \| 7 \| H \| -3.87568 \| -0.79968 \| -0.0013 \| |
|  | IM7 | \| Tag \| Symbol \| X \| Y \| Z \| \| --- \| --- \| --- \| --- \| --- \| \| 1 \| Pt \| 1.337321 \| -0.37559 \| -0.1175 \| \| 2 \| Pt \| -0.51905 \| 1.374607 \| 0.006544 \| \| 3 \| Pt \| -1.23095 \| -0.99651 \| 0.049198 \| \| 4 \| C \| 3.235594 \| -0.03694 \| 0.637725 \| \| 5 \| H \| 3.09156 \| 0.282019 \| 1.678407 \| \| 6 \| H \| 3.841026 \| 0.692196 \| 0.093548 \| \| 7 \| H \| 3.731183 \| -1.02447 \| 0.596794 \| \| 8 \| H \| 2.111703 \| 0.076274 \| -1.37834 \| |
|  | TS5 | \| Tag \| Symbol \| X \| Y \| Z \| \| --- \| --- \| --- \| --- \| --- \| \| 1 \| Pt \| 1.292585 \| -0.40648 \| -0.08081 \| \| 2 \| Pt \| -0.51082 \| 1.393061 \| 0.002164 \| \| 3 \| Pt \| -1.21575 \| -0.99529 \| 0.036566 \| \| 4 \| C \| 3.376208 \| 0.046944 \| 0.407957 \| \| 5 \| H \| 3.20966 \| 0.635169 \| 1.314663 \| \| 6 \| H \| 4.135974 \| 0.54345 \| -0.20738 \| \| 7 \| H \| 3.720158 \| -0.97102 \| 0.640938 \| \| 8 \| H \| 2.527327 \| 0.189825 \| -0.91374 \| |
|  | IM8 | \| Tag \| Symbol \| X \| Y \| Z \| \| --- \| --- \| --- \| --- \| --- \| \| 1 \| Pt \| 1.197757 \| -0.53307 \| -0.02035 \| \| 2 \| Pt \| -0.39886 \| 1.435422 \| -0.00078 \| \| 3 \| Pt \| -1.29361 \| -0.91095 \| 0.011252 \| \| 4 \| C \| 3.857651 \| 0.06197 \| 0.084979 \| \| 5 \| H \| 3.481092 \| -0.09275 \| 1.103579 \| \| 6 \| H \| 4.341898 \| 1.042241 \| 0.039349 \| \| 7 \| H \| 4.558047 \| -0.736 \| -0.17737 \| \| 8 \| H \| 3.0605 \| 0.085345 \| -0.7054 \| |


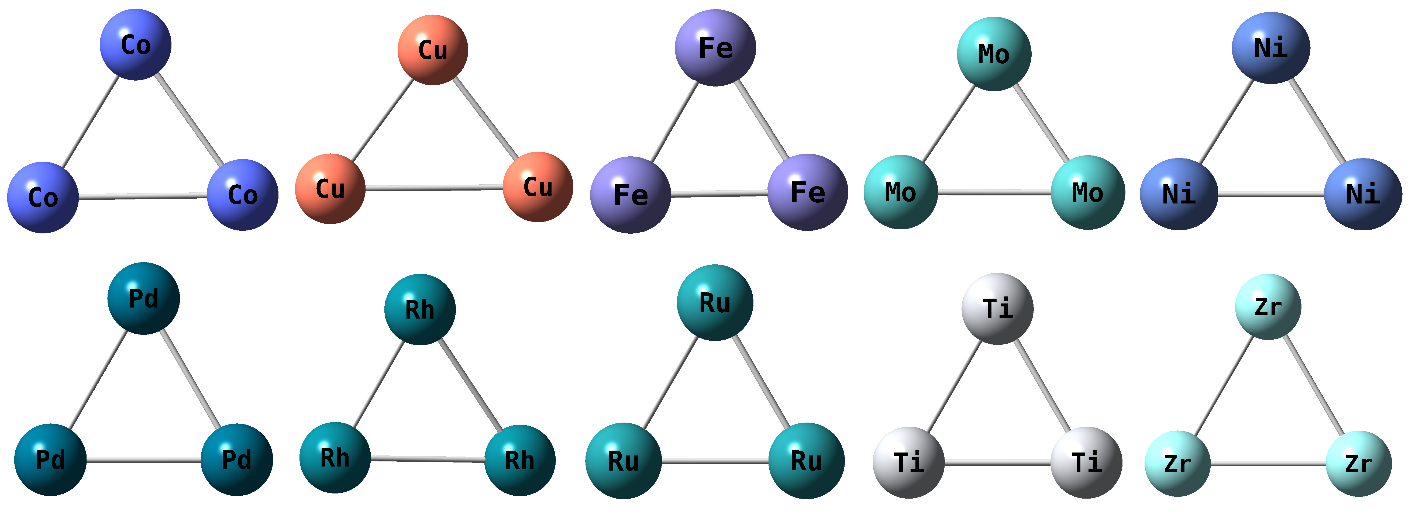


**Figure S1.** The catalysts employed for the estimation of adsorption energies of guaiacol.


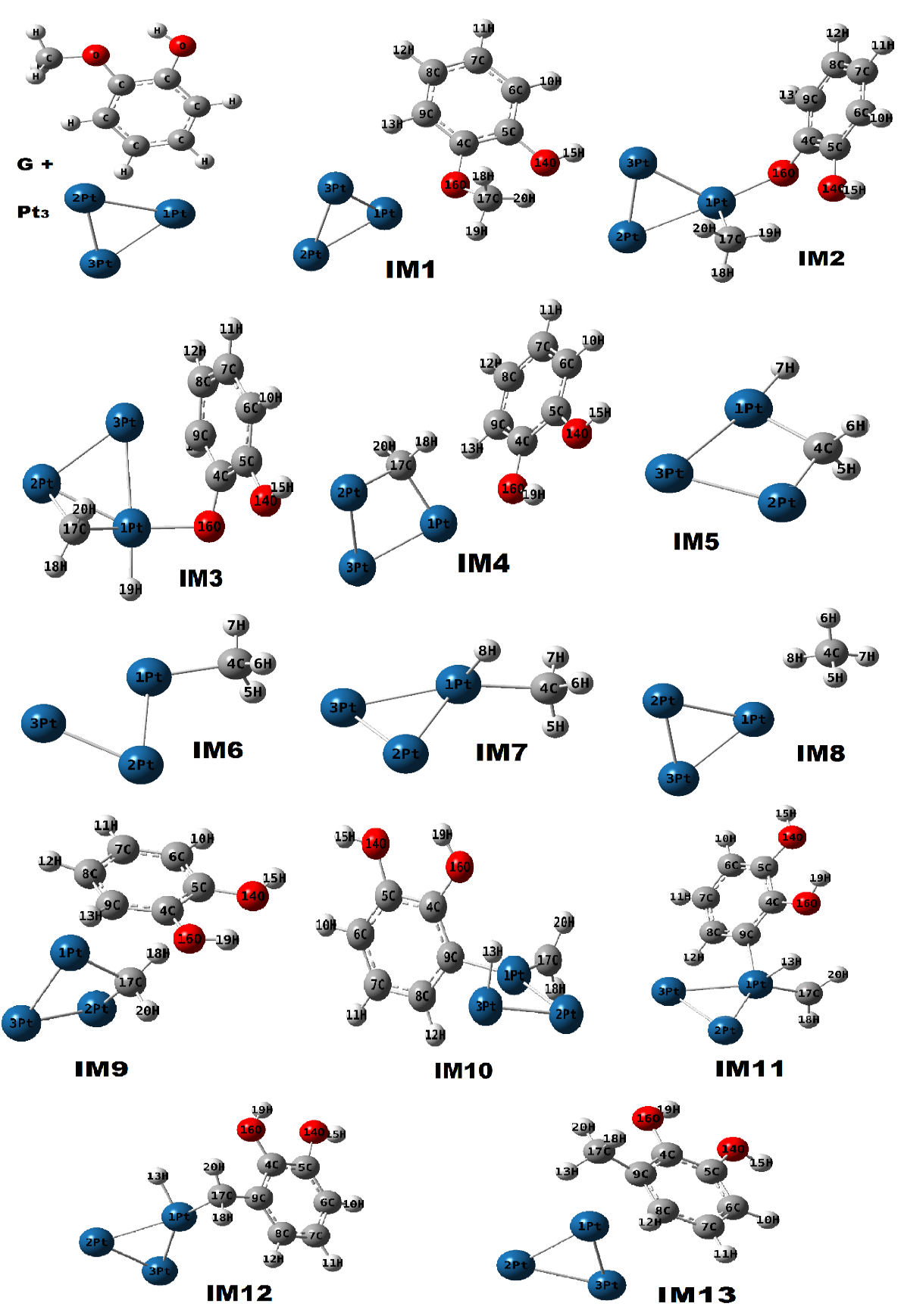


**Figure S2.** B3LYP/Gen optimized adsorbed intermediates in the conversion process of guaiacol into catechol and methyl catechol reaction over Pt_3_ catalyst cluster. IM1, IM8, and IM13 are co-adsorbed guaiacol, methane, and methylcatechol on to the catalyst cluster, respectively.


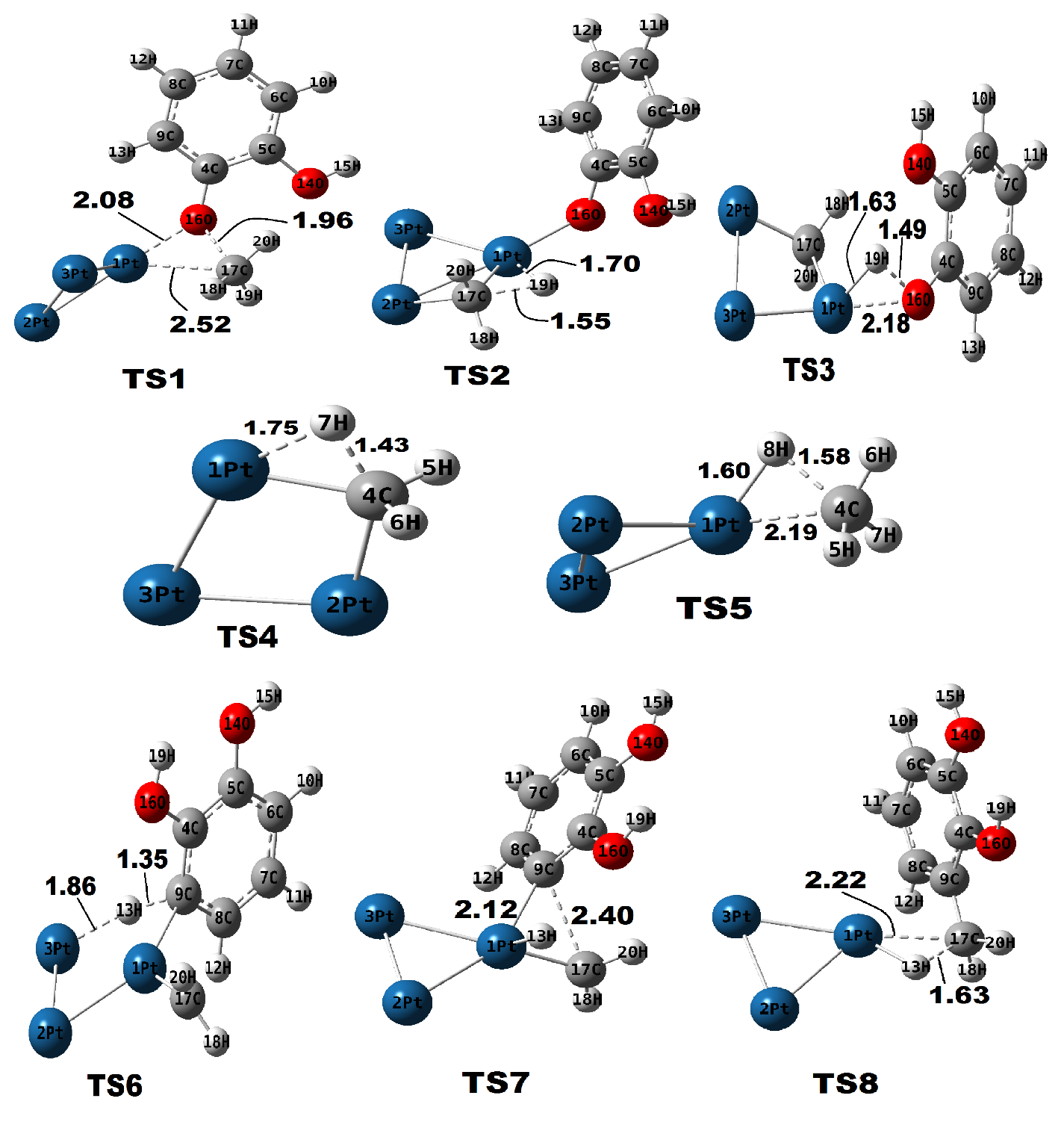


**Figure S3.** B3LYP/Gen optimized transition state structures in the conversion reaction of guaiacol into catechol and methylcatechol reaction over Pt_3_ catalyst cluster. The interatomic distances are reported in angstrom units.


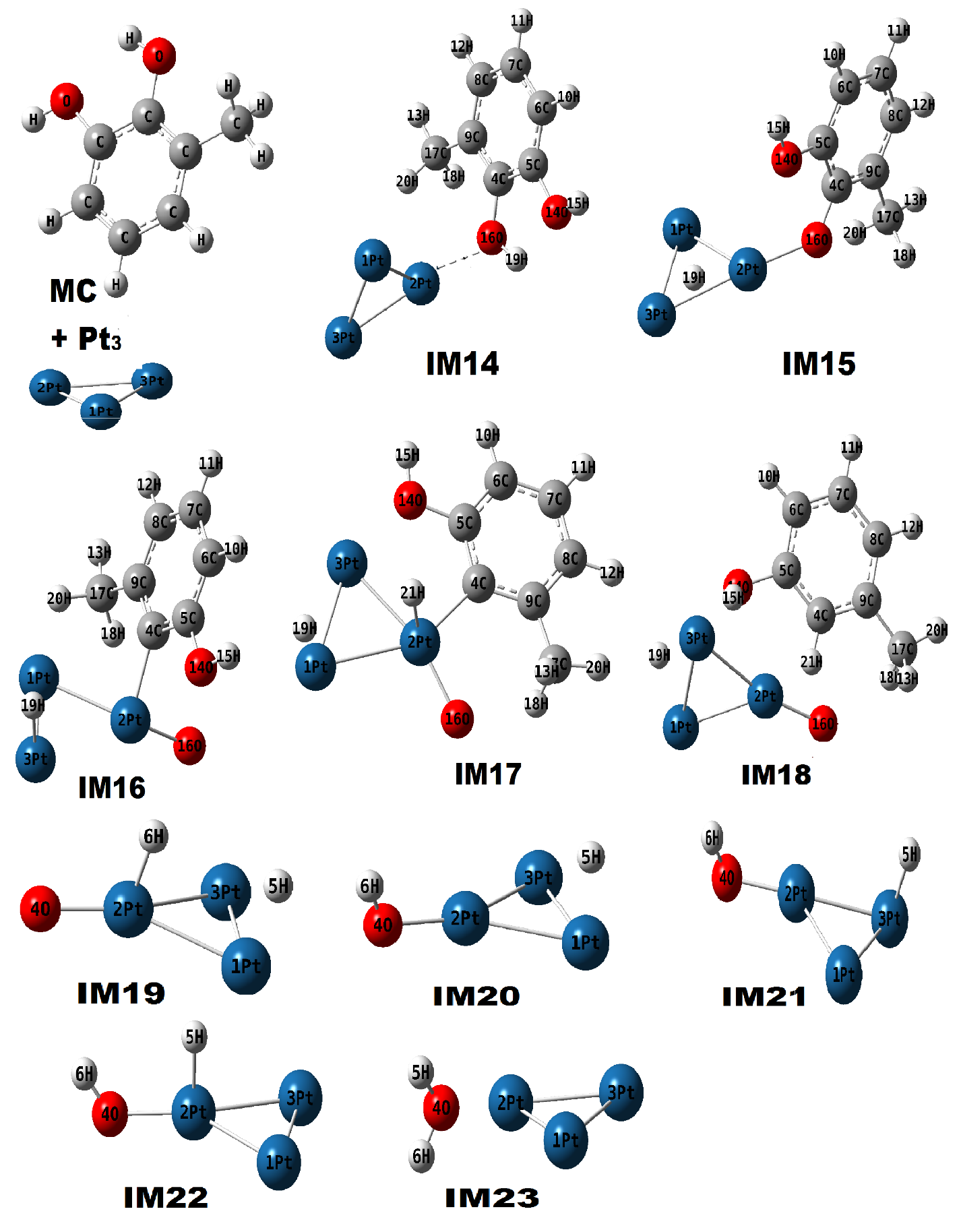


**Figure S4.** B3LYP/Gen optimized adsorbed intermediate structures for the formation of *m*-cresol from methylcatechol over Pt_3_ cluster. IM14 and IM23 are co-adsorbed methylcatechol and water species over Pt_3_ catalyst cluster, respectively.


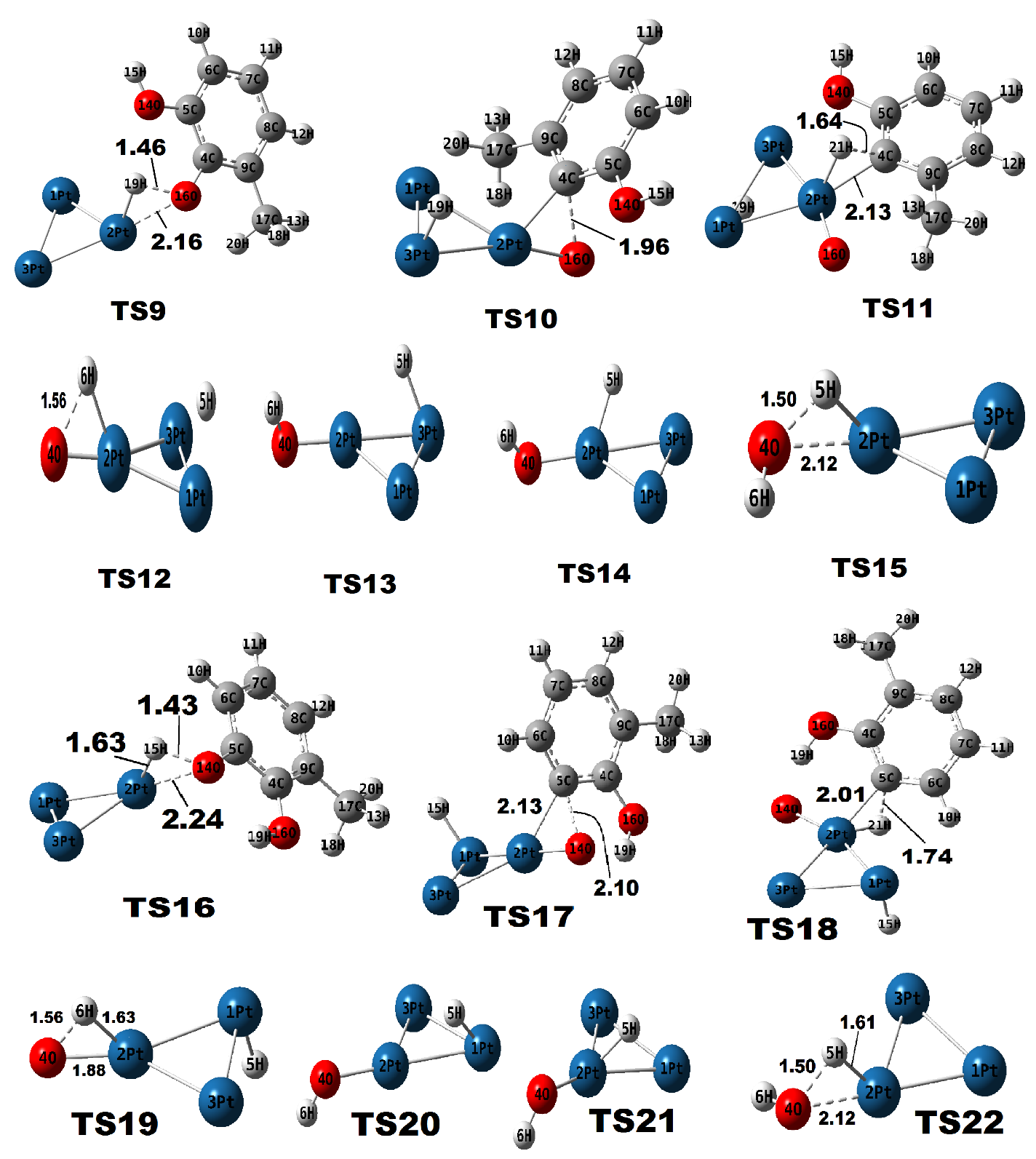


**Figure S5.** Transition state structures, optimized at B3LYP/Gen level of theory, for the formation of *m*-cresol and *o*-cresol from methylcatechol over Pt_3_ catalyst cluster. Interatomic distances are shown in angstrom units.


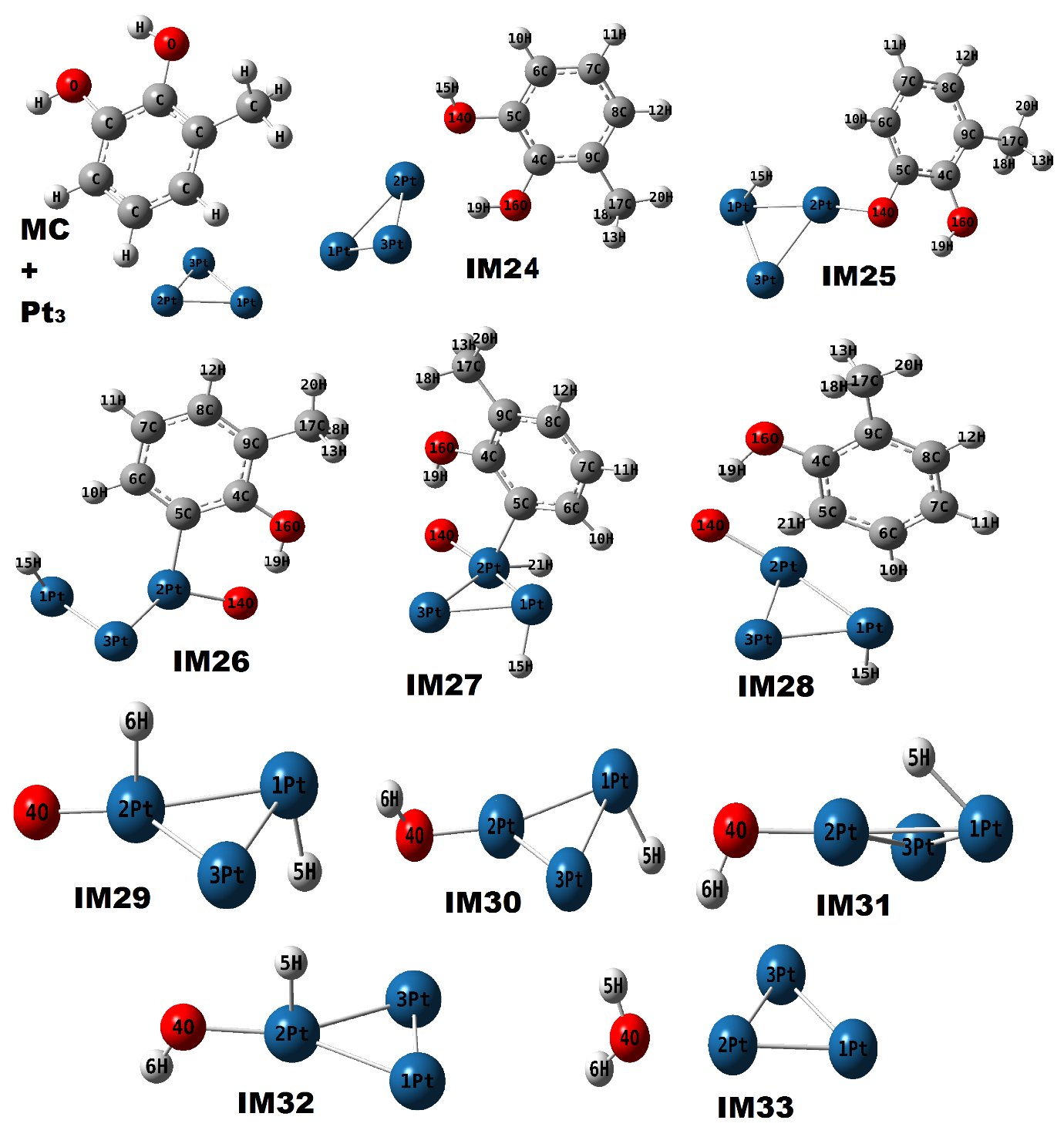


**Figure S6.** Intermediate structures, optimized using B3LYP/Gen level of theory, for the formation of *o*-cresol from methylcatechol in the presence of Pt_3_ catalyst cluster. IM24 and IM33 are adsorbed methylcatechol and water species over Pt_3_ catalyst cluster.
